# Down-regulation of cyclin-dependent kinase 5 attenuates p53-dependent apoptosis of hippocampal CA1 pyramidal neurons following transient cerebral ischemia

**DOI:** 10.1038/s41598-019-49623-x

**Published:** 2019-09-10

**Authors:** Bich Na Shin, Dae Won Kim, In Hye Kim, Joon Ha Park, Ji Hyeon Ahn, Il Jun Kang, Yun Lyul Lee, Choong-Hyun Lee, In Koo Hwang, Young-Myeong Kim, Sungwoo Ryoo, Tae-Kyeong Lee, Moo-Ho Won, Jae-Chul Lee

**Affiliations:** 1 Danchunok Company, Chuncheon, Gangwon 24210 Republic of Korea; 2 Department of Biochemistry and Molecular Biology, and Research Institute of Oral Sciences, College of Dentistry, Kangnung-Wonju National University, Gangneung, Gangwon 25457 Republic of Korea; 3 Famenity Biomedical Research Center, Famenity, Inc., Gwacheon, Gyeonggi 13837 Republic of Korea; 4 Department of Anatomy, College of Korean Medicine, Dongguk University, Gyeongju, Gyeongbuk 38066 Republic of Korea; 5 Department of Biomedical Science and Research Institute of Bioscience and Biotechnology, Hallym University, Chuncheon, Gangwon 24252 Republic of Korea; 6 Department of Food Science and Nutrition, Hallym University, Chuncheon, Gangwon 24252 Republic of Korea; 7 Department of Physiology, and Institute of Neurodegeneration and Neuroregeneration, College of Medicine, Hallym University, Chuncheon, Gangwon 24252 Republic of Korea; 8 Department of Pharmacy, College of Pharmacy, Dankook University, Cheonan, Chungcheongnam 31116 Republic of Korea; 9 Department of Anatomy and Cell Biology, College of Veterinary Medicine, and Research Institute for Veterinary Science, Seoul National University, Seoul, 08826 Republic of Korea; 10 Department of Molecular and Cellular Biochemistry, School of Medicine, Kangwon National University, Chuncheon, Gangwon 24341 Republic of Korea; 11 Department of Biological Sciences, College of Natural Sciences, Kangwon National University, Chuncheon, Kangwon 24341 Republic of Korea; 12 Department of Neurobiology, School of Medicine, Kangwon National University, Chuncheon, Gangwon 24341 Republic of Korea

**Keywords:** Stroke, Cell death in the nervous system

## Abstract

Abnormal activation of cyclin-dependent kinase 5 (Cdk5) is associated with pathophysiological conditions. Ischemic preconditioning (IPC) can provide neuroprotective effects against subsequent lethal ischemic insult. The objective of this study was to determine how Cdk5 and related molecules could affect neuroprotection in the hippocampus of gerbils after with IPC [a 2-min transient cerebral ischemia (TCI)] followed by 5-min subsequent TCI. Hippocampal CA1 pyramidal neurons were dead at 5 days post-TCI. However, treatment with roscovitine (a potent inhibitor of Cdk5) and IPC protected CA1 pyramidal neurons from TCI. Expression levels of Cdk5, p25, phospho (*p*)-Rb and *p*-p53 were increased in nuclei of CA1 pyramidal neurons at 1 and 2 days after TCI. However, these expressions were attenuated by roscovitine treatment and IPC. In particular, Cdk5, *p*-Rb and *p-*p53 immunoreactivities in their nuclei were decreased. Furthermore, TUNEL-positive CA1 pyramidal neurons were found at 5 days after TCI with increased expression levels of Bax, PUMA, and activated caspase-3. These TUNEL-positive cells and increased molecules were decreased by roscovitine treatment and IPC. Thus, roscovitine treatment and IPC could protect CA1 pyramidal neurons from TCI through down-regulating Cdk5, p25, and *p*-p53 in their nuclei. These findings indicate that down-regulating Cdk5 might be a key strategy to attenuate p53-dependent apoptosis of CA1 pyramidal neurons following TCI.

## Introduction

TCI occurs when blood flow to the brain is disrupted, resulting in oxygen and glucose deprivation of the tissues that can lead to “delayed neuronal death” in selectively vulnerable brain areas including the CA1 area of the hippocampus^1^. Clinically, brief ischemic events can induce ischemic tolerance by evoking the threshold of cerebral vulnerability, which is critical for neuroprotection^2^. It has been reported that transient ischemic damage can be prevented by ischemic preconditioning (IPC) in humans suffering transient ischemic attacks^3^ without leading to neuronal death^4,5^. Kitagawa *et al*. firstly introduced IPC as a strategy to attenuate ischemia-reperfusion injury in the brain using a gerbil model of TCI. Further studies have widely demonstrated IPC in other animal models of transient focal cerebral ischemia^6^. This phenomenon is called cerebral “ischemic tolerance”^7^, although mechanisms underlying brain ischemic tolerance have not been fully understood yet.

Cyclin-dependent kinase 5 (Cdk5), a proline-directed serine/threonine cyclin-dependent kinase family member, plays an important role in neuronal differentiation, axonal outgrowth, synaptogenesis, and memory formation^8^. Activation of Cdk5 can lead to translocation of Cdk5 from membrane to cytoplasm or nucleus^9^. Studies using rodents have also demonstrated that aberrant activity of Cdk5 primarily induces neuronal cell death during stroke^10,11^. To have normal function, Cdk5 has to bind to neuron-specific regulatory subunit protein p35 that is beneficial for neuronal development^12^. However, p35 is cleaved into p25 under a variety of pathological conditions^13^. Overexpression of p25 essentially activates Cdk5 and accesses various pathological substrates that trigger a cascade of neurotoxic pathways and culminate in neuronal death. For example, cerebral ischemic insults promote Ca^2+^ influx via calpain activation, which cleaves p35 to p25. The accumulation of p25 evokes a prolonged activation of Cdk5 in the brain. These alterations are responsible for neuronal death^14,15^. Furthermore, inhibition of the interaction between Cdk5 and p25 could decrease spinal injury in rats induced by acute spinal ischemia-reperfusion injury^16^. Therefore, inhibiting cleavage of p35 to p25 would be therapeutically beneficial against ischemic insults. In addition, up-regulation of Cdk5 is related to apoptosis in certain cell types^17,18^. However, mechanisms by which active Cdk5 can facilitate apoptosis are currently unclear, although it has been suggested that Cdk5 and its activators play important roles in the regulation of neuronal apoptosis following ischemic insults.

p53 (a tumor suppressor) plays an important role in regulating neuronal survival in the central nervous system^19^. However, in response to cellular stresses including DNA damage and hypoxia, p53 is a major inducer of apoptosis via up-regulation of many target genes in various cell types including neurons^20^. Moreover, biochemical hallmarks of apoptosis include caspase activation in animal models of ischemic insults^21^, and caspases have been identified as critical components in p53-induced cell death pathway^22^. Importantly, Cdk5 activity is related to increased p53 expression and activation^23^. However, whether this process occurs in IPC-induced neurons suffering from a subsequent TCI is currently unclear. Various possible explanations of neuroprotective effects of IPC against a subsequent ischemic damage have been proposed.

To the best of our knowledge, expression patterns of Cdk5, p53, and apoptosis-related proteins in IPC-induced brain following a subsequent TCI have not been reported yet. Thus, the objective of this study was to investigate effects of inhibiting Cdk5 and IPC on a subsequent ischemic insult. Changes in expressions of Cdk5, p35/p25, phospho (*p*)-p53, Bax, Bcl-2, PUMA, and caspase-3 in the hippocampus after roscovitine (a potent inhibitor of Cdk5) treatment and IPC following a subsequent TCI were determined using gerbil as a good animal model of TCI^24^.

## Results

### Roscovitine- and IPC-mediated neuroprotection against TCI

#### Cresyl violet-positive (CV^+^) cells

**Figure 1:**
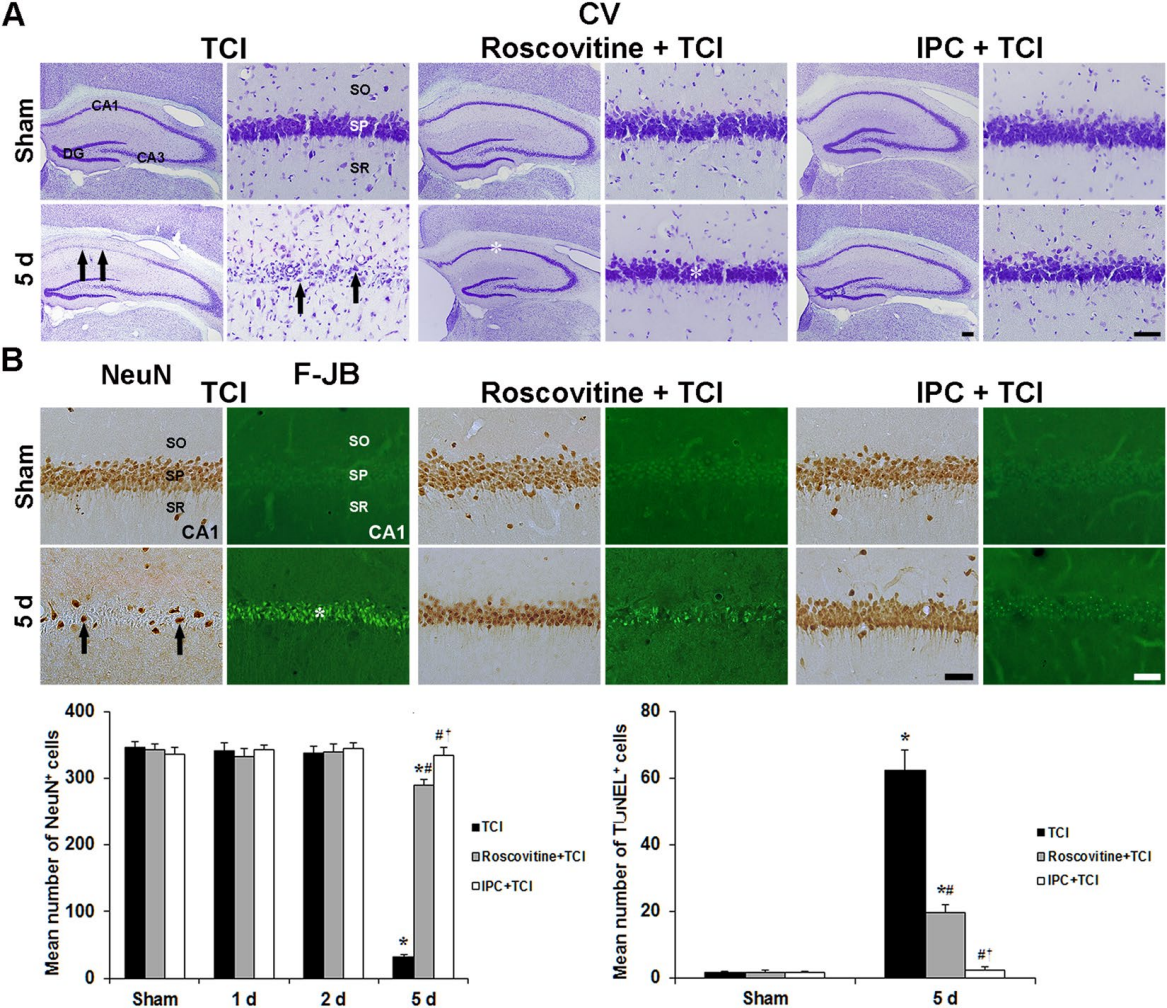
Roscovitine- and IPC-mediated neuroprotection against TCI. (**A**) CV staining in the hippocampus of the TCI (1^st^ and 2^nd^ columns), roscovitine + TCI (3^rd^ and 4^th^ columns) and IPC + TCI (5^th^ and 6^th^ columns) groups. CV^+^ CA1 pyramidal neurons (arrows) are damaged 5 days after TCI; however, CV^+^ CA1 pyramidal neurons (asterisks) in the roscovitine + TCI and IPC + TCI groups are similar to those in the sham group. Scale bar = 800 µm (1^st^, 3^rd^ and 5^th^ columns), 50 µm (2^nd^, 4^th^ and 6^th^ columns). (**B**) NeuN immunohistochemistry (1^st^, 3^rd^ and 5^th^ columns) and F-J B histofluorescence staining (2^nd^, 4^th^ and 6^th^ columns) in the CA1 area of the TCI (1^st^ and 2^nd^ columns), roscovitine + TCI (3^rd^ and 4^th^ columns) and IPC + TCI (5^th^ and 6^th^ columns) groups. In the TCI group, a few NeuN^+^ (arrows) and many F-J B^+^ (asterisk) CA1 pyramidal neurons are detected 5 days after TCI. In the roscovitine + TCI and IPC + TCI groups, many NeuN^+^ pyramidal neurons are observed in the CA1 area; F-J B-positive cells are lower than those in the TCI group. SO, stratum oriens; SP, stratum pyramidale; SR, stratum radiatum. Scale bar = 50 µm. Quantitative graphs for numbers of NeuN^+^ (left) and F-J B^+^ (right) CA1 pyramidal neurons. The bars are reported as means ± SEM from three independent experiments (*n* = 7, ^*^P < 0.05 vs. sham group; ^#^*P* < 0.05 vs TCI group; ^†^*P* < 0.05 vs roscovitine + TCI group).

Roscovitine- and IPC-induced neuronal survival by using CV staining in the gerbil hippocampal CA1 area after TCI is shown in Fig. 1A. In the sham group, CV^+^ cells in the stratum pyramidale of the CA1 area, which are named CA1 pyramidal neurons, were large in size, pyramidal or round in shape (Fig. 1A). In the TCI group, the morphology and numbers of CV^+^ CA1 pyramidal cells were not changed until 3 days after TCI (data not shown). At 5 days after TCI, CV^+^ CA1 pyramidal neurons were significantly damaged and hardly shown; however, pyramidal neurons in the CA2/3 area were intact (Fig. 1A).

In the roscovitine + sham group, the distribution pattern and morphology of CV^+^ CA1 pyramidal neurons were not different from the sham group (Fig. 1A). In the roscovitine + TCI groups, the distribution pattern and morphology of CV^+^ CA1 pyramidal neurons were similar to those in the sham groups (Fig. 1A).

In the IPC + sham group, the distribution pattern and morphology of CV^+^ CA1 pyramidal neurons were also not different from those in the sham group (Fig. 1A). In the IPC + TCI groups, the distribution pattern and morphology of CV^+^ CA1 pyramidal neurons were also like those in the sham group (Fig. 1A).

#### Neuronal nuclei^+^ (NeuN^+^) and Fluoro-Jade B^+^ (F-J B^+^) cells

Roscovitine- and IPC-mediated neuroprotection in the CA1 area was examined by NeuN immunohistochemistry and F-J B histofluorescence staining (Fig. 1B,C). In the sham group, CA1 pyramidal neurons were easily stained with NeuN, and no F-J B^+^ neurons were found in the CA1 area (Fig. 1B). In the TCI group, NeuN^+^ CA1 pyramidal neurons were significantly reduced (8.9 ± 4.3% of the sham group) and abundant F-J B^+^ CA1 pyramidal neurons were shown 5 days after TCI (Fig. 1B).

In the roscovitine + sham group, the distribution of NeuN^+^ CA1 pyramidal neurons was not different from that in the sham group, and any F-J B^+^ CA1 pyramidal neurons were not detected (Fig. 1B). In the roscovitine + TCI group, NeuN^+^ CA1 pyramidal neurons were well protected by roscovitine treatment (83.5 ± 5.2% of the sham group), and the number of F-J B^+^ cells was 20.7 ± 5.1% % lower than that in the 5 days after TCI (Fig. 1B).

In the IPC + sham and IPC + TCI groups, findings from NeuN immunohistochemistry and F-J B histofluorescence staining were not different from those in the sham group (Fig. 1B).

### Roscovitine- and IPC-mediated suppression of Cdk5 expression and its translocation after TCI

#### Cdk5 levels

**Figure 2:**
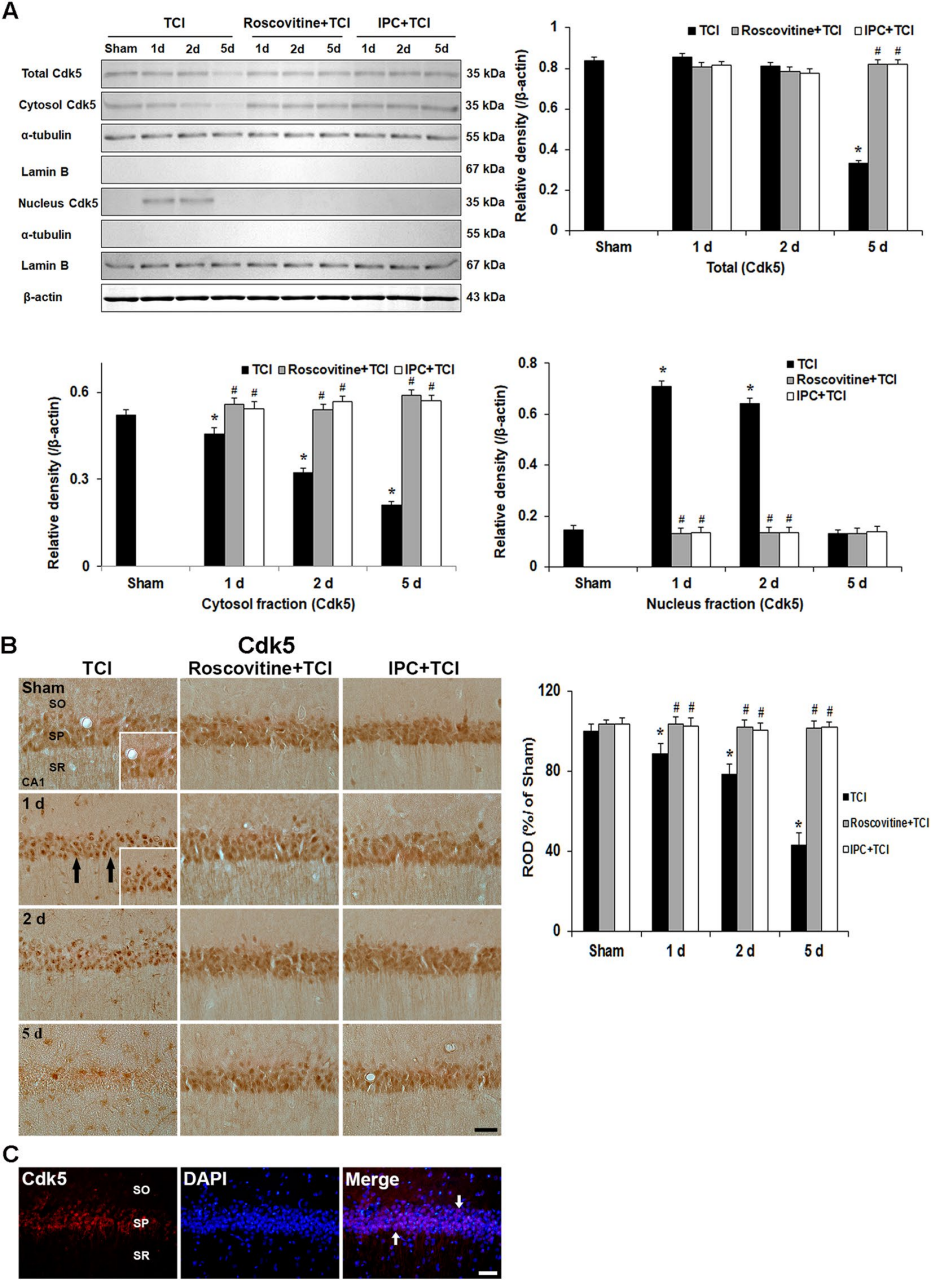
Effects of roscovitine and IPC on Cdk5 expression and its translocation after TCI. (**A**) Western blots of Cdk5 levels in the CA1 area of the TCI, roscovitine + TCI and IPC + TCI groups at sham, 1, 2 and 5 days after TCI. α-Tubulin, lamin B and β-actin densitometric values are used to standardize for cytosol and nucleus protein loading, respectively. Relative band intensity of total cytosol and nucleus Cdk5 level is measured by densitometer. Molecular weight is indicated as kDa on the right side of the immunoblots. The bars are reported as means ± SEM from three independent experiments (*n* = 7, ^*^P < 0.05 vs. sham group; ^#^*P* < 0.05 vs TCI group; ^†^*P* < 0.05 vs roscovitine + TCI group). (**B**) Immunohistochemistry of Cdk5 in the CA1 area of the TCI (left column), roscovitine + TCI (middle column), and IPC + TCI (right column) groups at sham, 1, 2 and 5 days after TCI. Cdk5 immunoreactivity is translocated into nuclei (arrows) in CA1 pyramidal neurons of the TCI group 1 and 2 days after TCI and hardly detected 5 days after TCI. In the roscovitine + sham, IPC + sham, roscovitine + TCI and IPC + TCI groups, Cdk5 immunoreactivity in CA1 pyramidal neurons is similar to that in the sham group. SO, stratum oriens; SP, stratum pyramidale; SR, stratum radiatum. Scale bar = 50 µm. Quantitative graph of Cdk5 immunoreactivity in CA1 pyramidal neurons. A ratio of the ROD was calibrated as %, with the sham group designated as 100%. The bars are reported as means ± SEM from three independent experiments (*n* = 7, ^*^P < 0.05 vs. sham group; ^*#*^*P* < 0.05 vs TCI group; ^†^*P* < 0.05 vs roscovitine + TCI group). (**C**) Double immunofluorescence staining for Cdk5 (red), DAPI (blue) and merged images at 1 day after TCI. Cdk5^+^ immunofluorescence in the SP are colocalized with DAPI^+^ nuclei (white arrows). Scale bar = 50 μm.

To identify whether Cdk5 activation is required for delayed neuronal death in the CA1 area after TCI, we examined levels of Cdk5 proteins in the CA1 area with or without roscovitine treatment and IPC after TCI (Fig. 2A). Cdk5 was immunoprecipitated from nucleus and cytosol extracts of the CA1 area (Fig. 2A). Cdk5 protein in the CA1 area of the sham group appeared throughout the nucleus and cytosol fractions, and the cdk5 protein level was significantly increased in the cytosol fraction compared with that in the nucleus fraction (Fig. 2A).

In the TCI groups, Cdk5 protein levels increased significantly in the nucleus fraction with a concomitant reduction in the cytosol fraction 1 and 2 days after TCI, and Cdk5 protein levels in both fractions were significantly decreased 5 days after TCI compared with those in the sham group (Fig. 2A).

Cdk5 protein levels in the roscovitine + TCI group were not altered compared with that in the roscovitine + sham group (Fig. 2A).

In the IPC + TCI group, levels of Cdk5 protein were similar to that in the sham group (Fig. 2A).

#### Cdk5 immunoreactivity

We examined change in Cdk5 immunoreactivity in the CA1 area in all experimental groups (Fig. 2B). Cdk5 immunoreactivity was detected in the cytoplasm of the CA1 pyramidal neurons in the sham group (Fig. 2B). One and 2 days after TCI, Cdk5 immunoreactivity in the cytoplasm of CA1 pyramidal neurons was not shown, instead, Cdk5 immunoreactivity was observed only in nuclei of the CA1 pyramidal neurons (Fig. 2B). Five days after TCI, Cdk5 immunoreactivity in CA1 pyramidal neurons was barely observed (Fig. 2B).

In the roscovitine + sham group, Cdk5 immunoreactivity in CA1 pyramidal neurons was similar to that in the sham group. In the roscovitine + TCI group, the pattern of Cdk5 immunoreactivity did not change at any time after TCI compared with that in the roscovitine + sham group (Fig. 2B).

In the IPC + sham and IPC + TCI groups, Cdk5 immunoreactivity was also like that in the roscovitine + sham and roscovitine + TCI groups (Fig. 2B).

In addition, one day after TCI, we confirmed that the Cdk5 immunoreactivity was found in DAPI stained nuclei of the CA1 pyramidal neurons (Fig. 2C).

#### Roscovitine- and IPC-mediated expressions of p35 and p25 levels after TCI

**Figure 3:**
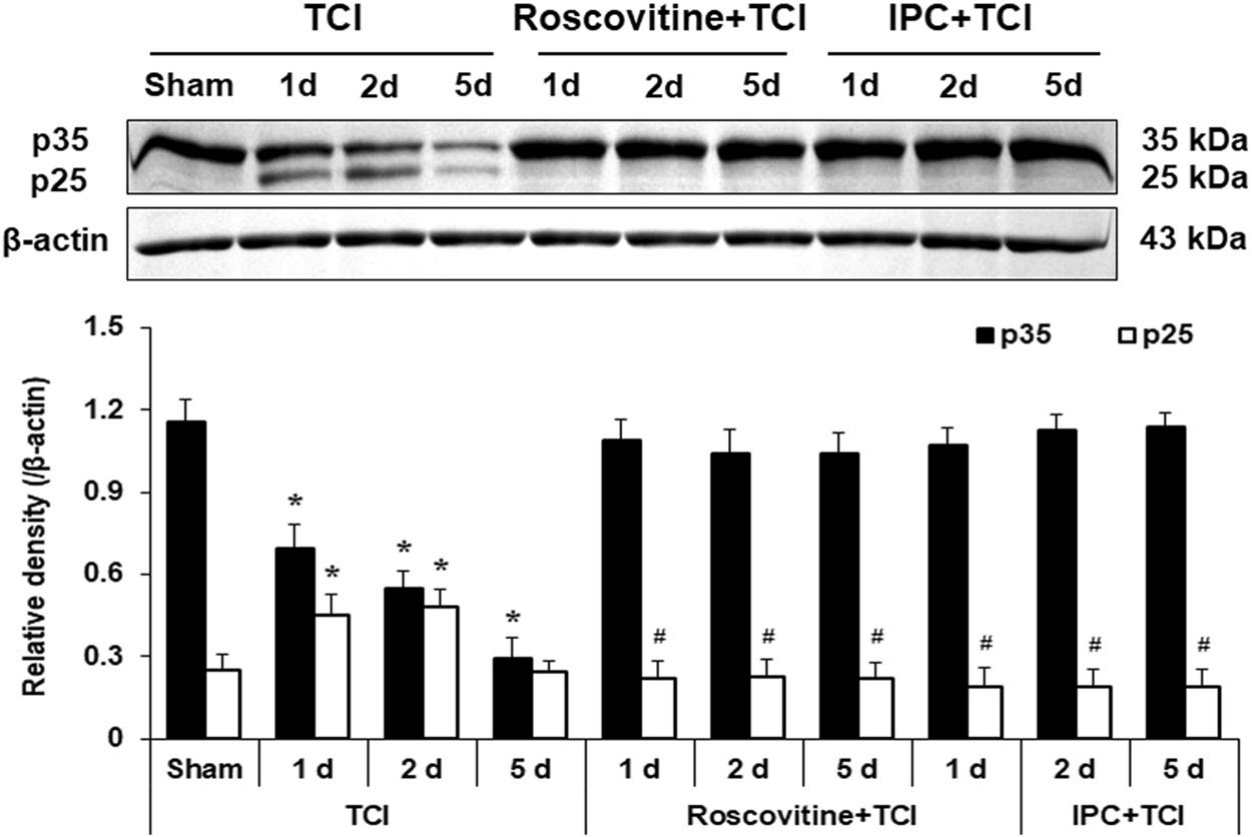
Effects of roscovitine and IPC on levels of p35/p25 proteins in the TCI, roscovitine + TCI and IPC + TCI groups at sham, 1, 2 and 5 days after TCI. β-actin is used as a protein loading control. Relative band intensity of p35/p25 levels was measured by densitometer. Levels of p25 protein in the roscovitine + TCI and IPC + TCI groups are significantly low compared with those in the TCI group. Molecular weight is indicated as kDa on the right side of the immunoblots. The bars are reported as means ± SEM from three independent experiments (*n* = 7, ^*^P < 0.05 vs. sham group; ^#^*P* < 0.05 vs TCI group; ^†^*P* < 0.05 vs roscovitine + TCI group).

Subsequently, western blots were performed to investigate temporal expression patterns of relevant proteins such as p35 and p25 with roscovitine- and IPC-mediated neuroprotection in tissues of the CA1 area (Fig. 3). High level of p35 protein was detected in the CA1 area of the sham group (Fig. 3). In the TCI group, the level of p35 protein was slightly decreased 1 day and 2 days after TCI compared to those in the sham group, and intensely decreased 5 days after TCI (Fig. 3). On the contrary, p25 protein level was low in the sham group, and the level markedly increased 1 and 2 days after TCI (Fig. 3).

In the roscovitine + TCI group, p35 protein levels were not changed at any times after TCI compared with those in the roscovitine + sham group (Fig. 3). The level of p25 in the roscovitine + sham group was similar to that in the sham group (data not shown). In the roscovitine + TCI group, p25 levels were also not changed at any times after TCI compared with that in the roscovitine + sham group (Fig. 3).

In the IPC + TCI group, change pattern of p35 protein levels was like that in the roscovitine + TCI group (Fig. 3). In the IPC + TCI group, levels of p25 protein were higher than those in the roscovitine + TCI group from 1 day to 5 days after TCI (Fig. 3).

### Roscovitine- and IPC-mediated suppressions of Rb phosphorylation after TCI

#### Rb levels

**Figure 4:**
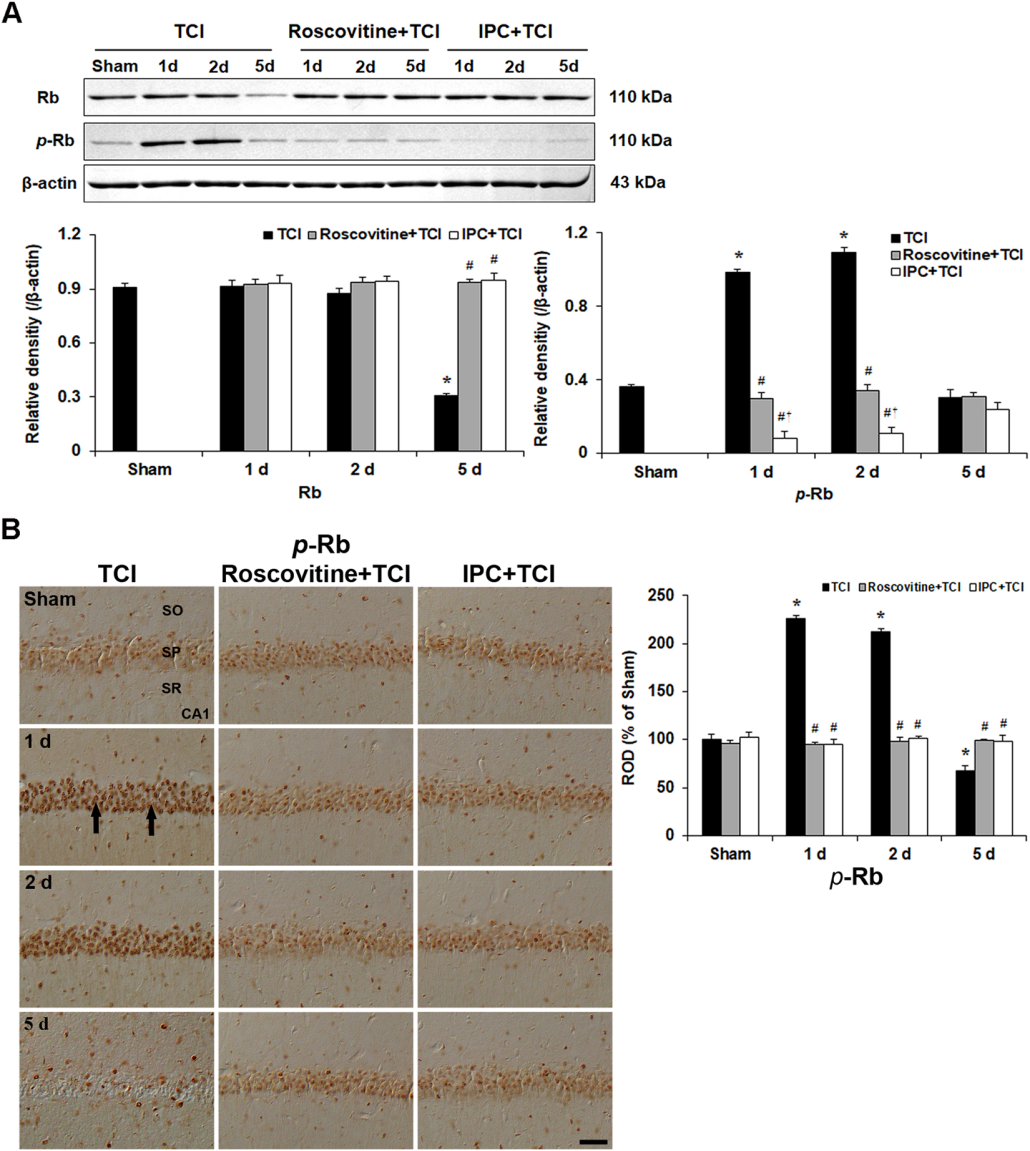
Effects of roscovitine and IPC on Rb expression after TCI. (**A**) Western blots of total Rb and *p*-Rb in the CA1 area of the TCI, roscovitine + TCI and IPC + TCI groups at sham, 1, 2 and 5 days after TCI. β-actin is used as a protein loading control. Relative band intensity of Rb and *p*-Rb level is measured by densitometer. *p*-Rb level is significantly low in the roscovitine + TCI and IPC + TCI groups compared with the TCI group. Molecular weight is indicated as kDa on the right side of the immunoblots. The bars are reported as means ± SEM from three independent experiments (*n* = 7, ^*^P < 0.05 vs. sham group; ^#^*P* < 0.05 vs TCI group; ^†^*P* < 0.05 vs roscovitine + TCI group). (**B**) *p*-Rb immunoreactivity in the CA1 area of the TCI (left column), roscovitine + TCI (middle column) and IPC + TCI (right column) groups at sham, 1, 2 and 5 days after TCI. *p*-Rb immunoreactivity in the TCI group is very strong in nuclei (arrows) of CA1 pyramidal neurons 1 and 2 days after TCI. In the roscovitine + TCI and IPC + TCI groups, the pattern of *p*-Rb immunoreactivity in CA1 pyramidal neurons was similar to that in the sham group. SO, stratum oriens; SP, stratum pyramidale; SR, stratum radiatum. Scale bar = 50 µm. Quantitative graph of *p*-Rb immunoreactivity in CA1 pyramidal neurons. A ratio of the ROD was calibrated as %, with the sham group designated as 100%. The bars are reported as means ± SEM from three independent experiments (*n* = 7, ^*^P < 0.05 vs. sham group; ^#^*P* < 0.05 vs TCI group; ^†^*P* < 0.05 vs roscovitine + TCI group).

Previous some data have suggested that the phosphorylation of Rb protein could be mediated through Cdk5 pathway. Therefore, we carried out Rb and *p-*Rb western blotting to demonstrate the p25-Cdk5 activity (Fig. 4A).

The level of *p-*Rb protein was weakly observed in the CA1 area of the sham group, significantly enhanced 1 and 2 days after TCI and markedly decreased 5 days after TCI (Fig. 4A), which coincides with Cdk5/p25 protein activity after TCI.

In the roscovitine + TCI group, the level of *p-*Rb protein was not changed at any times after TCI compared with that in the sham group (Fig. 4A).

In the IPC IPC + TCI group, levels of *p-*Rb protein were similar to that in the sham group (Fig. 4A).

#### p-Rb immunoreactivity

We examined cellular distribution of *p-*Rb protein in CA1 pyramidal neurons with or without roscovitine treatment and IPC, respectively, after TCI (Fig. 4B).

**Figure 5:**
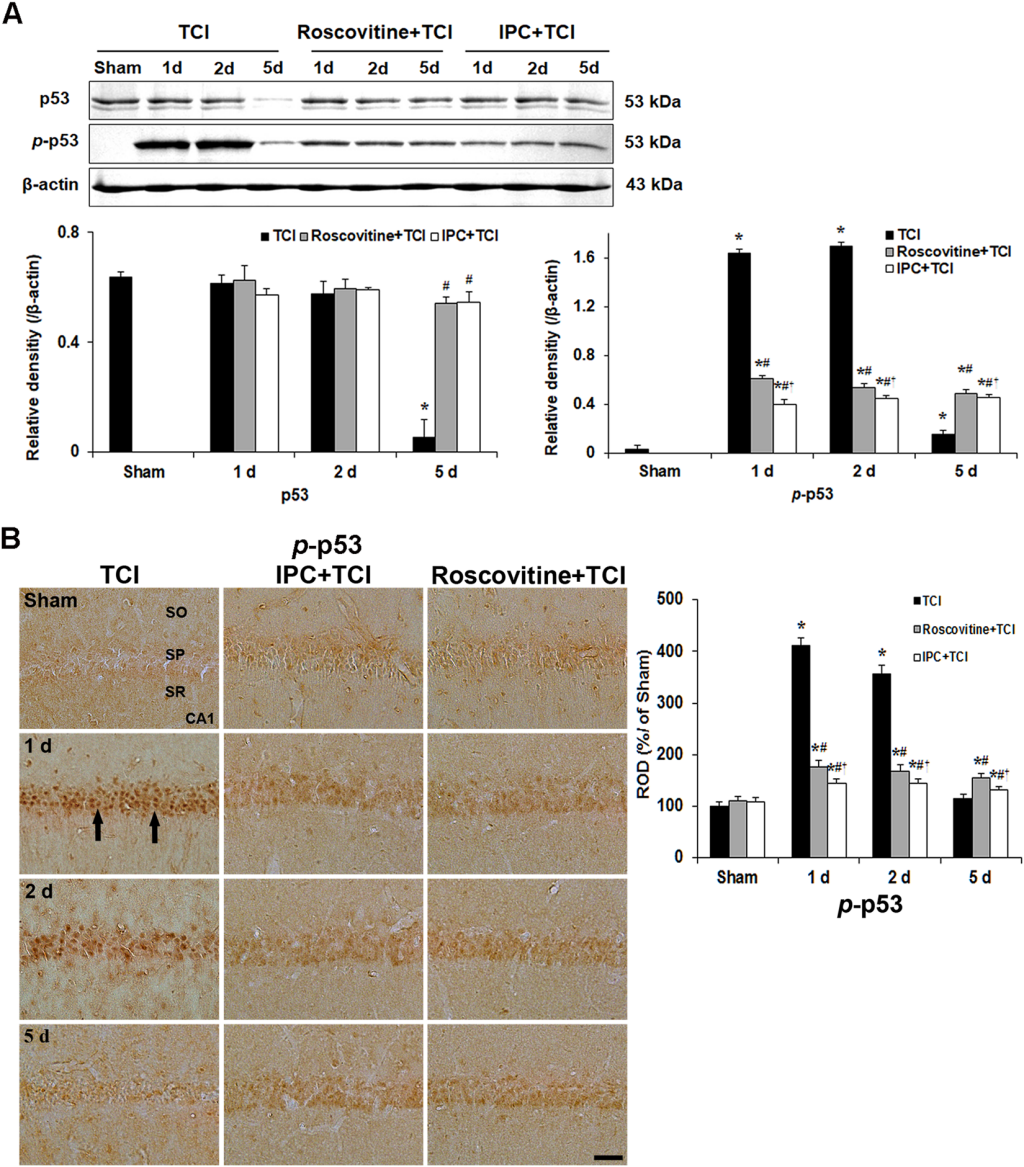
Effects of roscovitine and IPC on p53 phosphorylation and its translocation after TCI. (**A**) Western blots of p53 and *p*-p53 in the CA1 area of the TCI, roscovitine + TCI and IPC + TCI groups at sham, 1, 2 and 5 days after TCI. β-actin is used as a protein loading control. Relative band intensity of total p53 and *p*-p53 level is measured by densitometer. *p*-p53 levels are significantly low in the roscovitine + TCI and IPC + TCI groups compared with the TCI group. Molecular weight is indicated as kDa on the right side of the immunoblots. The bars are reported as means ± SEM from three independent experiments (*n* = 7, ^*^P < 0.05 vs. sham group; ^#^*P* < 0.05 vs TCI group; ^†^*P* < 0.05 vs roscovitine + TCI group). (**B**) *p*-p53 immunoreactivity in the CA1 area of the TCI (left column), roscovitine + TCI (middle column) and IPC + TCI (right column) groups at sham, 1, 2 and 5 days after TCI. *p*-p53 immunoreactivity in the TCI group is very strong in nuclei (arrows) of CA1 pyramidal neurons 1 and 2 days after TCI. In the roscovitine + TCI and IPC + TCI groups, *p*-p53 immunoreactivity in CA1 pyramidal neurons is moderated 1 and 2 days after TCI, and the immunoreactivity is shown in both nuclei and cytoplasm. SO, stratum oriens; SP, stratum pyramidale; SR, stratum radiatum. Scale bar = 50 µm. Quantitative graph of *p*-p53 immunoreactivity in CA1 pyramidal neurons. A ratio of the ROD was calibrated as %, with the sham group designated as 100%. The bars are reported as means ± SEM from three independent experiments (*n* = 7, ^*^P < 0.05 vs. sham group; ^#^*P* < 0.05 vs TCI group; ^†^*P* < 0.05 vs roscovitine + TCI group).

*p-*Rb immunoreactivity in CA1 pyramidal neurons of the sham group was very weak (Fig. 5B). In the TCI group, many CA1 pyramidal neurons showed strong *p-*Rb immunoreactivity in their nuclei 1 and 2 days after TCI; however, *p-*Rb immunoreactivity in CA1 pyramidal neurons was markedly decreased at 5 days after TCI (Fig. 4B).

In the roscovitine + sham and roscovitine + TCI groups, *p-*Rb immunoreactivity weakly appeared in nuclei of CA1 pyramidal neurons and was similar to that in the sham group (Fig. 4B).

In the IPC + sham and IPC + TCI groups, the pattern of *p-*Rb immunoreactivity in CA1 pyramidal neurons was similar to that in the roscovitine + sham and roscovitine + TCI groups (Fig. 4B).

### Roscovitine- and IPC-mediated suppression of *p-*p53 expression and its translocation after TCI

#### p-p53 levels

As described in above, Cdk5 was upregulated in nuclei of CA1 pyramidal neurons after TCI. This finding needed to investigate whether Cdk5 was relevant to the phosphorylation of p53 protein after TCI. Based on this hypothesis, we examined the phosphorylation of p53 protein in CA1 pyramidal neurons with or without roscovitine and IPC, respectively, after TCI (Fig. 5A).

Western blot showed that the level of *p-*p53 protein in the sham group was weak, significantly enhanced 1 and 2 days after TCI and markedly decreased 5 days after TCI (Fig. 5A). This coincided with Cdk5 protein level/kinase activity after TCI.

In the roscovitine + TCI group, the level of *p-*p53 protein was significantly lower at all times after TCI compared with that in the TCI group (Fig. 5A).

In the IPC + TCI group, the change pattern of *p-*p53 protein was similar to that in the roscovitine + TCI group; however, the level of *p-*p53 protein was lower compared with that in the roscovitine + TCI group (Fig. 5A).

#### p-p53 immunoreactivity

Subsequently, we examined cellular distribution of *p-*p53 protein in CA1 pyramidal neurons with or without roscovitine and IPC, respectively, after TCI (Fig. 5B).

*p-*p53 immunoreactivity was very weak in CA1 pyramidal neurons of the sham group (Fig. 5B). In the TCI group, many CA1 pyramidal neurons showed strong *p*-p53 immunoreactivity in their nuclei 1 day after TCI, and similar *p-*p53 immunoreactivity was maintained 2 days after TCI; however, *p*-p53 immunoreactivity in CA1 pyramidal neurons was very weak at 5 days after TCI (Fig. 5B).

In the roscovitine + sham group, weak *p-*p53 immunoreactivity was shown in nuclei of CA1 pyramidal neurons (Fig. 5B). In the roscovitine + TCI group, increased *p-*p53 immunoreactivity was observed in both cytoplasm and nuclei of CA1 pyramidal neurons 1 day after TCI, and the pattern was similar 2 days after TCI (Fig. 5B). Five days after TCI, *p-*p53 immunoreactivity in CA1 pyramidal neurons area was also not changed compared with that at 2 days after TCI (Fig. 5B).

In the IPC + sham group, *p-*p53 immunoreactivity in CA1 pyramidal neurons was not different from that in the roscovitine + sham group (Fig. 5B). In the IPC + TCI group, the pattern of *p-*p53 immunoreactivity in CA1 pyramidal neurons was similar to that in the roscovitine + TCI group; however, the immunoreactivity was lower compared with that in the roscovitine + TCI group (Fig. 5B).

#### Roscovitine- and IPC-mediated suppression of Bax, PUMA and active caspase-3 levels after TCI

**Figure 6:**
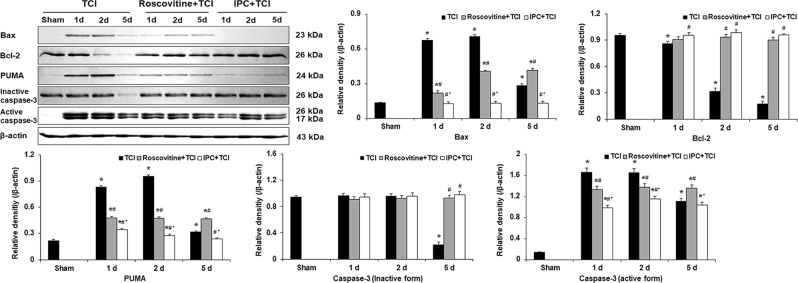
Effects of roscovitine and IPC on Bax, Bcl-2, PUMA and caspase-3 (inactive and active form) levels in the TCI, roscovitine + TCI and IPC + TCI groups at sham, 1, 2 and 5 days after TCI. β-actin is used as a protein loading control. Relative band intensity of Bax, Bcl-2, PUMA and caspase-3 (inactive and active form) levels is measured by densitometer. Molecular weight is indicated as kDa on the right side of the immunoblots. The bars are reported as means ± SEM from three independent experiments (*n* = 7, ^*^P < 0.05 vs. sham group; ^#^*P* < 0.05 vs TCI group; ^†^*P* < 0.05 vs roscovitine + TCI group).

To explore a possible pattern of the death of CA1 pyramidal neurons after TCI, we examined levels of Bax, Bcl-2, PUMA and caspase-3 proteins in the CA1 area with or without roscovitine and IPC after TCI (Fig. 6).

Western blot analyses showed that levels of Bax, PUMA and active caspase-3 proteins were significantly increased 1 day and 2 days after TCI (Fig. 6). This coincided with the pattern of p53 levels at the same point in time after TCI. On the other hand, Bcl-2 level was significantly increased 1 day after TCI and gradually decreased with time after TCI (Fig. 6).

In the roscovitine + TCI group, levels of Bax, PUMA and caspase-3 proteins were significantly decreased, and Bcl-2 levels were markedly increased compared to the TCI group from 1 day to 5 days after TCI (Fig. 6).

In the IPC + TCI group, the pattern of levels of Bax, Bcl-2, PUMA and caspase 3 proteins was similar to that in the roscovitine + TCI group; however, levels of Bax, PUMA and caspase 3 proteins were higher than those in the roscovitine + TCI group from 1 day to 5 days after TCI, but, Bcl-2 levels were not changed compared with that in the roscovitine + TCI group (Fig. 6).

### Roscovitine- and IPC-mediated suppression of apoptosis following TCI

**Figure 7:**
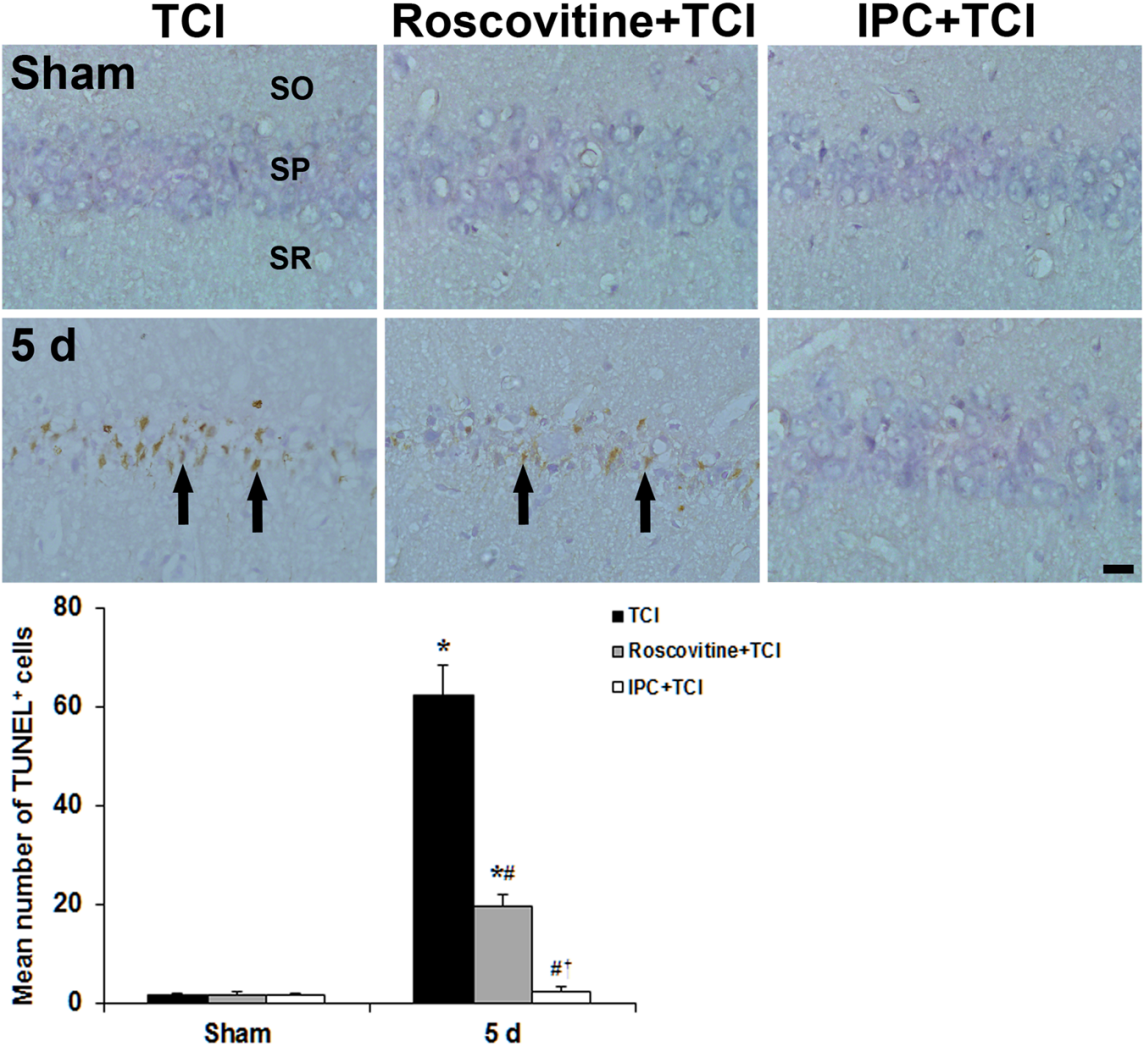
Effect of roscovitine and IPC on apoptosis of CA1 pyramidal neuroins using TUNEL staining in the TCI (left column), roscovitine + TCI (middle column), and IPC + TCI (right column) groups at sham and 5 days after TCI. Many TUNEL^+^ CA1 pyramidal neurons (arrows) are found in the stratum pyramidale (SP) of the TCI group 5 days after TCI. However, TUNEL^+^ cells are significantly low in the roscovitine + TCI and IPC + TCI groups compared with the TCI group, respectively. SO, stratum oriens; SR, stratum radiatum. Scale bar = 50 µm. The quantitative graph is shown the percentage of TUNEL^+^ cells in the SP. The bars are reported as means ± SEM from three independent experiments (*n* = 7, ^*^P < 0.05 vs. sham group; ^#^*P* < 0.05 vs TCI group; ^†^*P* < 0.05 vs roscovitine + TCI group).

To investigate whether Cdk5 was relevant to neuronal apoptosis after TCI, we carried out TUNEL staining in the CA1 area with or without roscovitine and IPC after TCI (Fig. 7).

In the sham group, no TUNEL^+^ cells were found in the CA1 area; however, a significant increase of TUNEL^+^ CA1 pyramidal neurons was observed in the stratum pyramidale 5 days after TCI (Fig. 7).

In the roscovitine + sham group, no TUNEL^+^ CA1 pyramidal neurons cells were found, and, in the roscovitine + TCI group, TUNEL^+^ cells (31.4 ± 2.35% of the TCI group) were found 5 days after TCI (Fig. 7).

In the IPC + sham group, also, TUNEL^+^ CA1 pyramidal neurons were not shown in the CA1 stratum pyramidale, and few TUNEL^+^ CA1 pyramidal neurons (3.76 ± 1.2% of the TCI group) were found in the IPC + TCI group 5 days after TCI (Fig. 7).

## Discussion

IPC cannot lead to neuronal damage/death in ischemic brains because it can induce neuronal tolerance to a longer and/or severer subsequent transient ischemia^25^. In the present study, IPC was induced by subjecting gerbils to a 2 min of transient brain ischemia. We found that CA1 pyramidal neurons were not killed in the hippocampus following TCI. This means that IPC effectively protected neurons from TCI. In this regard, remarkable protection induced by IPC might be an attractive strategy to develop potential therapeutics^26^.

Neuronal death in the brain following cerebral ischemia or stoke is associated with inappropriate activation of Cdk5^27^. For instance, Cdk5 activity is increased rat brain following focal and global cerebral ischemia^10,11^. In stroke-affected patients, the expression of phosphorylated Cdk5 is substantially increased in damaged neurons, but not in healthy brain neurons^28^. On the other hand, in a rat model of hypoxia/ischemia injury, inhibition of Cdk5 activity after hypoxia/ischemic insult can dramatically reduce infarction and displays functional recovery^29^. It has been reported that roscovitine, a potent inhibitor of Cdk5 in neurons, can decrease transient ischemia-induced Tau hyperphosphorylation^30^, microglia proliferation, and excessive production of various inflammatory cytokines^31^ in ischemic brain regions. In addition, roscovitine has been suggested as a neuroprotective agent in rodent models of focal cerebral ischemia^30,32^. Moreover, systemic administration of roscovitine can significantly decrease total infarct volume when it is administered either before or after ischemia onset^32^. In present study, treatment with roscovitine effectively protected CA1 pyramidal neurons from TCI. However, little is known about the change of Cdk5 expression after TCI. O’Hare *et al*. have reporetd that activation of Cdk5 can lead to translocation of Cdk5 from intracellular membrane to cytoplasm or nucleus^33^. In the present study, Cdk5 immunoreactivity was localized predominantly to the periphery of the cytoplasm of CA1 pyramidal neurons in the sham group, meaning that Cdk5 was localized to the intracellular membrane of CA1 pyramidal neurons, based on the report of O’Hare *et al*.^33^. In the TCI group, Cdk5 was found only within the nuclei of CA1 pyramidal neurons at 1–2 days after TCI while its protein level in the nuclear fraction was significantly increased. However, Cdk5 expression in the nuclei of neurons was abolished by treatment with roscovitine or IPC. Instead, Cdk5 was shown throughout the cytoplasm. Our present finding strongly suggests that neuronal cell death after cerebral ischemia might be associated with Cdk5 activation.

For neuronal death, it has been reported that nuclear Cdk5 activity can enhance neuronal cell death in *in vitro* models of excitotoxicity^33^ and in a Parkinson’s disease model induced by 1-methyl-4-phenyl 1,2,3,6 tetrahydropyridine^34^. It is clear that nuclear Cdk5 activity can facilitate neuronal cell death in cerebral ischemia^35,36^. However, Cdk5 in the cytoplasm must play dual roles in death/survival of cells. For instance, it has been reported that Cdk5 within cytoplasm can mediate excitotoxicity by phosphorylating peroxiredoxin 2 under ischemic conditions^37^. In contrast, O’Hare *et al*. have reported that cytoplasmic Cdk5 can promote survival following DNA damage and suggested that cytoplasmic Cdk5 displays a pro-survival role under normal conditions^33^. In this regard, our present finding of Cdk5 immunoreactivity in the cytoplasm of CA1 pyramidal neurons in both roscovitine + TCI and IPC + TCI groups indicates that the roscovitine- and IPC-mediated cytoplasmic localization of Cdk5 following TCI might play a role in neuronal survival. Our results strongly suggest that cytoplasmic activity of Cdk5 might be normal or pathogenic while nuclear activity of Cdk5 might be pathogenic.

It is well known that Cdk5 must bind to p35 to perform its function^12^. However, p35 is cleaved into p25 under a variety of pathological conditions^13^. Hayashi *et al*. have reported that immunoreactivities of both Cdk5 and p35 are increased in penumbra region following transient focal cerebral ischemia and that p25 expression is increased in infarcted region in rats^38^. In particular, some researchers have demonstrated that accumulation of p25 after transient cerebral ischemia can activate Cdk5 which induces hippocampal CA1 cell death by directly phosphorylating NMDA receptors^15^. In the present study, p25 was increased while p35 was down-regulated in CA1 pyramidal neurons following TCI. In detail, p25 level of the nuclear fraction was significantly increased at 1–2 days after TCI while its level was significantly down-regulated at 1–2 days after TCI by roscovitine treatment and IPC. It is reasonable to understand that upregulation of p25 is relevant to abnormal activation of Cdk5 which might be associated with neuronal death following ischemia-reperfusion injury. On the other hand, in this study, roscovitine treatment and IPC significantly decreased the elevation of p25 expression and simultaneously inhibited the down-regulation of p35 following TCI. Taken together, our present findings suggest that TCI can cause up-regulation of p25 by increasing the cleavage of p35, resulting in over-activation of Cdk5 which might be a key process that leads to neuronal cell death following TCI.

Some researchers have reported that roscovitine can protect neurons in models of spinal ischemic damage, stroke, and traumatic brain injury, showing that roscovitine can significantly decrease the expression of Cdk5/p25 complex^36,37^. In addition, Menn *et al*. have reporetd that permanent focal cerebral ischemia can induce an increase in Cdk5/p25 activity that leads to irreversible cell damage and cell death while systemic administration of roscovitine can prevent such increase of Cdk5/p25 activity^32^. This provides direct evidence linking roscovitine-mediated neuroprotection to Cdk5-dependent mechanisms. Therefore, inhibiting cleavage of p35 to p25 might have potential as a therapeutic strategy for cerebral ischemia injury^36^. Some data have suggested that phosphorylation of retinoblastoma (Rb) protein could be mediated through Cdk5 pathway. Rb phosphorylation at Ser807/811 has been reported as a substrate of aberrant Cdk5/p25 in injured neurons^39^. Phosphorylation of Rb protein is an early event in p25/Cdk5-induced neurotoxicity^40^. In addition, p25/Cdk5 is detected in nuclei of degenerating neurons^41^. It can interact with nuclear substrates^17,23^. In agreement with these findings, our experiments showed nuclear localization of p25 and Cdk5. Moreover, pRb level was significantly increased at 1–2 days after TCI while Rb phosphorylation disappeared after roscovitine treatment and IPC. These data suggest that TCI can induce aberrant Cdk5/p25 activation which then leads to increased phosphorylation of Rb protein.

In cerebral ischemia-reperfusion injury, p53 has been identified as one of critical inducers of neuronal death. For instance, compounds that can cause DNA damage are known to induce p53 activation in cultured neurons whereas NF-κB activity is decreased significantly^42^. In addition, pifithrin-α, a p53 inhibitor, can preserve NF-κB activity and protect neurons from transient brain ischemic injury^43^. Furthermore, neuroprotection and functional recovery after ischemia-reperfusion insults have been demonstrated via knockout or inhibition of p53 in animal models of ischemia-reperfusion^44,45^. In our present study, immunohistochemical analysis showed strong p53 immunoreactivity in nuclei of CA1 pyramidal neurons at 1 and 2 days after TCI while roscovitine treatment and IPC significantly decreased *p-*p53 expression in the nuclei and protected CA1 pyramidal neurons from TCI. This finding is supported by studies showing that IPC can lead to a decrease of p53 level and neuronal resistance to subsequent ischemia^46^. Our present results strongly suggest that the detrimental effect of p53 following TCI is associated with p53 expression in nuclei of damaged neurons.

Here, we will discuss the relationship between Cdk5 and p53 in cell damage. Zhang *et al*. have reporetd that p53 and Cdk5 levels are increased concomitantly in apoptotic PC12 cells and that Cdk5/p25 can effectively phosphorylates recombinant p53. In addition, transient transfection of Cdk5/p25 into cells can increase p53 level that is functionally active^23^. Lee and Kim have reported that increased Cdk5 activity can induce neuronal death by regulating p53 expression in cerebral cortical neurons in a mouse model of DNA damage and that inhibition of Cdk5 activity by roscovitine treatment can dramatically block p53 phosphorylation in damaged neurons^47^. Leker *et al*. have reporetd that, although p53 has a short half-life and its activity maintains at low level under no stress, resident p53 translocation into nuclei and binding to its specific DNA sites are early events in p53-induced apoptosis in neurons following ischemic insults. They have suggested that prevention of p53 translocation could reduce brain damage^48^.

Accumulating evidence has suggested that phosphorylation pattern of p53 at various sites is critical for the regulation of cell death which depends on cell type and extracellular stimuli. p53 protein can be phosphorylated at a minimum of 20 Ser/Thr residues within its N- and C-terminal regions^49–51^. DNA damage can induce phosphorylation of p53 at Ser15 and Ser20, leading to reduced interaction of p53 with its negative regulator HDM2 which consequently leads to p53 accumulation^52,53^. Homeodomain-interacting protein kinase-2 (HIPK2) can phosphorylate Ser46 in response to UV radiation and drive an apoptotic response^54^. Dual specificity protein phosphatase 26 (DUSP26) can regulate p53-mediated apoptosis in high-risk neuroblastoma (NB) and contributes to chemoresistance by inhibiting p53 function. Shi *et al*.^55^ have demonstrated that DUSP26 can physically bind to p53, dephosphorylate p53 at Ser37, and cause inhibition of downstream p53 signaling. In addition, Lee *et al*.^56^ have reported that Cdk5 can phosphorylate p53 on Ser15, Ser33 and Ser46 *in vitro* and that increased Cdk5 activity in the nucleus can mediate phosphorylation events in response to genotoxic and oxidative stresses. In the present study, we showed that p53 phosphorylation at Ser37 was significantly enhanced in nuclei of CA1 pyramidal neurons at 1 and 2 days after TCI, coinciding with changes in Cdk5 level and immunoreactivity. Thus, the change of *p-*p53 expression at Ser37 in cellular nucleus might occur as a consequence of Cdk5 kinase activity. Although there are many studies on phosphorylation of p53, physiological roles of p53 phosphorylation at specific sites remain unclear. Furthermore, *in vivo* studies on protein kinases that can modulate the phosphorylation state and function of p53 have not been reported yet. Our findings strongly suggest that p53 is a direct substrate for Cdk5, although expression patterns of Cdk5 and p53 in IPC-induced brain following a subsequent TCI remain unclear.

p53 can mediate apoptosis through transcriptional activation of pro-apoptotic genes including Bax and PUMA^57^. PUMA can inhibit the function of anti-apoptotic Bcl-2 and induce the release of pro-apoptotic Bax^58^. Niizuma *et al*. have shown that PUMA is up-regulated to bound to Bax in CA1 pyramidal neurons after global brain ischemia and that PUMA upregulation is inhibited by pifithrin-α. They have suggested that PUMA is controlled by p53 transcriptional pathway after global cerebral ischemia^59^. In addition, Ren *et al*. have reporeted that PUMA can initiate apoptosis via Bax after neutralizing all members of anti-apoptotic Bcl-2 like molecules^60^. In the present study, Bax and PUMA levels were increased while Bcl-2 level was decreased in the CA1 area after TCI. These changes were inhibited by roscovitine treatment or IPC. Moreover, in the present study, proteolytic activation of the caspase-3 was significantly increased in the CA1 area at 1–2 days after TCI while the increase of caspase-3 was inhibited by roscovitine treatment or IPC. It has been reported that caspase-3 is an important component in p53-induced apoptosis^61^ and that caspase-3 activation is involved in apoptotic neuronal death in the brain following cerebral ischemia^62,63^. Furthermore, it has been demonstrated that genetic deletion and pharmacological inhibition of caspases can exert neuroprotective effects against cerebral ischemic insults^64^. Taken together, our results suggest that IPC can prevent TCI-mediated apoptosis in CA1 pyramidal neurons through p53-mediated PUMA signaling pathway.

In the present study, TUNEL^+^ cells were found in CA1 pyramidal neurons at 5 days after TCI. However, TUNEL^+^ cells were significantly decreased in roscovitine + TCI and IPC + TCI groups compared to those in the TCI group. Sandal *et al*. have reported that Cdk5 activation can occur by activation of upstream caspase-3. They argued that Cdk5 activity needed cleavage of pro-enzyme caspase-3 to its active form in cAMP-induced apoptosis of leukemia cells^65^. Taken together, our present finding suggests that Cdk5-dependent p53 regulation can promote apoptosis via caspase-3. This encourages us to speculate that Cdk5 is one of key factors that facilitate neuronal apoptosis via p53 activation after ischemic insults.

In summary, our present findings showed that roscovitine treatment and IPC clearly protected CA1 pyramidal neurons from a subsequent severer TCI and that roscovitine- and IPC-mediated neuroprotection were closely associated with down-regulation of Cdk5 and p25. In addition, down-regulation of Cdk5 by roscovitine treatment and IPC might be a key factor in attenuating p53-dependent apoptosis after TCI. Our results strongly suggest that down-regulation of Cdk5 is critical in neuroprotection as well as IPC-mediated tolerance against various ischemic insults.

## Methods

### Experimental groups

Male Mongolian gerbils (*Meriones unguiculatus*) were obtained from the Experimental Animal Center, Kangwon National University, Chuncheon, South Korea. They were 6-month old and 65–75 g in body weight. Animal handling and care went after the guidelines of current international laws and policies from the NIH Guide for the Care and Use of Laboratory Animals (The National Academies Press, 8th Ed., 2011). The experimental protocols were approved by Institutional Animal Care and Use Committee (IACUC) of Kangwon National University (approval no. KW-160802-1). As previously described^66^, gerbils were divided into 6 groups (n = 14 at each point in time in each group): (1) sham TCI-operated group (sham group) was given no ischemia; (2) TCI-operated group (TCI group) was given a 5 min of TCI; (3) Roscovitine (a potent inhibitor of Cdk5)-treated and sham TCI-operated group (roscovitine + sham group) was intraperitoneally injected roscovitine; (4) Roscovitine-treated and TCI-operated group (roscovitine + TCI group) was subjected to TCI after roscovitine treatment; (5) IPC-treated and sham TCI-operated group (IPC + sham group) was subjected to IPC, which was induced by a 2 min of transient ischemia, and given no TCI; and (6) IPC + TCI group was subjected to TCI following IPC.

### Treatment of roscovitine

To examine whether roscovitine inhibited Cdk5 in the hippocampus induced by TCI, roscovitine (40 mg/kg; Sigma–Aldrich, St. Louis, MO, USA, R7772) was intraperitoneally injected 60 min prior to sham or TCI operation.

### Surgery of IPC and TCI

Transient ischemia was developed according to our published procedure^67^. In brief, the experimental animals were anesthetized with a mixture of 2.5% isoflurane in 33% oxygen and 67% nitrous oxide. Ischemic insults were induced by bilateral common carotid artery occlusion. A 2 min and a 5 min of occlusion were carried out for IPC and TCI, respectively. Normothermic (37 ± 0.5 °C) condition was maintained before, during and after the ischemic surgery until the animals completely recovered from anesthesia.

### Sacrifice of animals

IPC paradigm has been proven to be very effective at protection of neurons from transient ischemic injury in this ischemic model^67^. The gerbils in all groups were given recovery times of 1, 2 and 5 days after ischemia, because pyramidal neurons in the hippocampal CA1 area do not die until about 3 days and begin to die at about 4 days after a 5 min of TCI^68^.

### Western blot analyses

The nuclear and cytosolic fractions were prepared as previously described^69^, and western blot analyses for Cdk5, p35/25, *p*-Rb, *p*-p53, Bax, Bcl-2, PUMA and caspase-3 (inactive and active form) in the ischemic CA1 area (n = 7/group) were done according to our previous method^33^. Briefly, the tissues were homogenized, and protein levels in the supernatants were determined using a Micro BCA protein assay kit (Pierce Biotechnology, Inc., Rockford, IL, USA). The membranes were incubated with rabbit anti-Cdk5 (1:1,000 dilution, Santa Cruz Biotechnology Inc.), rabbit anti-p35/25 (1:1,000 dilution, Cell Signaling Technology, Danvers, MA), rabbit anti-Rb (1:1,000 dilution, Cell Signaling Technology), rabbit anti-Rb (phospho Ser807/811) (1:1,000 dilution, Cell Signaling Technology), rabbit anti-p53 (1:1,000 dilution, Abcam), rabbit anti-p53 (phospho Ser37) (1:1,000 dilution, Abcam), rabbit anti-Bax (1:1,500 dilution, Santa Cruz Biotechnology Inc.), mouse anti-Bcl-2 (1:1,500 dilution, Santa Cruz Biotechnology Inc.), mouse anti-PUMA (1:1,500 dilution, Santa Cruz Biotechnology Inc.), rabbit anti-caspase-3 (1:1,000 dilution, Abcam), rabbit anti-active caspase-3 (1:1,000 dilution, Abcam), rabbit anti-lamin B (1:1,500 dilution, Santa Cruz Biotechnology Inc.), mouse anti-α-tubulin (1:1,000 dilution, Abcam), and mouse anti-β-actin (1:2,000 dilution, Sigma-Aldrich). Finally, the immunoreactive bands were visualized by an ECL kit (Pierce Biotechnology).

### Tissue processing for histology

According to our published procedure for histology^66^, in brief, animals (n = 7 in each group) were anesthetized with pentobarbital sodium (30 mg/kg; JW Pharmaceutical, Seoul, Korea) and perfused transcardially with 4% paraformaldehyde and their hippocampal tissues were serially cut into 30-μm coronal sections.

### CV staining

To examine cellular distribution and damage, CV staining was performed by our published procedure^66^. In brief, cresyl violet acetate (Sigma–Aldrich) was dissolved at 1.0% (w/v), and glacial acetic acid (0.28%) was added to this solution. The sections were stained and dehydrated.

### F-J B histofluorescence staining

To examine neuronal degeneration, F-J B (a high affinity fluorescent marker for the localization of neurodegeneration) staining was performed according to our published procedure^70^. Briefly, the sections were immersed in a solution containing 1% sodium hydroxide, transferred to a solution of 0.06% potassium permanganate and then a 0.0004% Fluoro-Jade B (Histochem, Jefferson, AR, USA) solution. After washing, the sections were placed on a slide warmer (approximately 50 °C), and examined using an epifluorescent microscope (Carl Zeiss, Göttingen, Germany) with blue (450–490 nm) excitation light and a barrier filter.

### Immunohistochemistry

Immunohistochemistry was carried out according to our published procedure^66^. In short, the sections were incubated with primary mouse anti-neuronal nuclei (NeuN, a neuron-specific soluble nuclear antigen) (diluted 1:1,000, Chemicon International, Temecula, CA, USA), rabbit anti-Cdk5 (diluted 1:100, Santa Cruz Biotechnology Inc. Santa Cruz, CA, USA), rabbit anti-Rb (phospho Ser807/811) (1:100 dilution, Cell Signaling Technology), and rabbit anti-p53 (phospho Ser37) (1:100, Abcam, Cambridge, UK). Theses sections were next incubated with secondary antibodies (Vector Laboratories Inc., Burlingame, CA) and developed using Vectastain ABC (Vector Laboratories Inc.). And they were visualized with 3,3’-diaminobenzidine.

### Double immunofluorescence staining

To confirm whether Cdk5 immunoreactivity is translocated to nuclei or not following TCI, the sections were assessed via DAPI staining at 1 day after the ischemic surgery. Cdk5 immunofluorescence staining was performed using rabbit anti-Cdk5 (diluted 1:100, Santa Cruz Biotechnology Inc.). The sections were incubated in the primary antibody overnight at room temperature. After washing 3 times for 10 min with PBS, these sections were then incubated in Cy3-conjugated horse anti-rabbit IgG (1:200; Jackson ImmunoResearch Laboratories Inc.) for 2 h at room temperature. They were then stained with DAPI (10 μM) for 10 min at room temperature in the dark, and then rinsed thoroughly in PBS. The immunoreactions were observed under the confocal MS (LSM510 META NLO, Carl Zeiss, Göttingen, Germany).

### TUNEL staining

TUNEL (terminal deoxynucleotidyl dUTP nick-end labeling) staining was carried out according to our published procedure^71^. In brief, frozen 8-μm coronal sections of the CA1 area were incubated in TUNEL reaction mixture according to kit instructions (Roche Molecular Biochemicals) and The TUNEL reaction incubated in converter-POD (Roche Molecular Biochemicals). Finally, they were treated with DAB-substrate solution.

### Data analysis

To count cells according to our published method^66^, in short, digital images from 15 sections per animal were taken using light microscope (AxioM1, Carl Zeiss) equipped with digital camera (Axiocam, Carl Zeiss) connected to a PC monitor. Cells were counted in a 200 × 200 µm square including the stratum pyramidale at the center of the CA1 area using an image analyzing system (software: Optimas 6.5, CyberMetrics, Scottsdale, AZ). Cell counts were obtained by averaging the counts from each animal. To analyze immunoreactivity, according to our previous method^66^, briefly, images of the immunoreactive structures were captured like the above-described method. The density of the immunoreactive structures was evaluated based on optical density (OD), which was obtained after the transformation of the mean gray level using the formula: OD = log (256/mean gray level). Background density in images was subtracted, and brightness and contrast were calibrated as % (relative optical density, ROD) using Adobe Photoshop version 8.0 and then analyzed using NIH Image J software. A ratio of the ROD was calibrated as %, with sham group designated as 100%. According to our published method^66^, results of western blot analyses were scanned and densitometric analyses for the quantification of the bands was done using Scion Image software (Scion Corp., Frederick, MD, USA). The expression rates of the target proteins were normalized through the corresponding expression rates of β-actin.

### Statistical analysis

Sample size was at least seven rats per group with an alpha error of 0.05 and a power of >80%, and the sample size was calculated with power calculator. All data are presented as mean ± S.E.M. A multiple-sample comparison was applied to test the differences between groups (ANOVA and the Tukey multiple range test as post hoc test using the criterion of the least significant differences). Statistical significance was considered at *P* < 0.05.

## Supplementary information

The uncut images of western blot
