# Supplementary material for: Down-regulation of cyclin-dependent kinase 5 attenuates p53-dependent apoptosis of hippocampal CA1 pyramidal neurons following transient cerebral ischemia
Source: Sci Rep. 2019 Sep 10;9:13032. doi: 10.1038/s41598-019-49623-x (PMC6737192; doi:10.1038/s41598-019-49623-x)

Down-regulation of cyclin-dependent kinase 5 attenuates p53-dependent apoptosis of hippocampal CA1 pyramidal neurons following transient cerebral ischemia

Bich Na Shin[1,#], Dae Won Kim[2,#], In Hye Kim[3], Joon Ha Park[4], Ji Hyeon Ahn[4], Il Jun Kang[5], Yun Lyul Lee[6], Choong-Hyun Lee[7], In Koo Hwang[8], Young-Myeong Kim[9], Sungwoo Ryoo[10], Tae-Kyeong Lee[11], Moo-Ho Won[11,*], Jae-Chul Lee[11,*]

[1]*Danchunok Company, Chuncheon, Gangwon, 24210 Republic of Korea*

[2]*Department of Biochemistry and Molecular Biology, and Research Institute of Oral Sciences, College of Dentistry, Kangnung-Wonju National University, Gangneung, Gangwon, 25457 Republic of Korea*

[3]*Famenity Biomedical Research Center, Famenity, Inc., Gwacheon, Gyeonggi, 13837 Republic of South Korea*

[4]*Department of Biomedical Science and Research Institute of Bioscience and Biotechnology, Hallym University, Chuncheon, Gangwon, 24252 Republic of Korea*

[5]*Department of Food Science and Nutrition, Hallym University, Chuncheon, Gangwon, 24252 Republic of Korea*

[6]*Department of Physiology, and Institute of Neurodegeneration and Neuroregeneration, College of Medicine, Hallym University, Chuncheon, Gangwon, 24252 Republic of Korea*

[7]*Department of Pharmacy, College of Pharmacy, Dankook University, Cheonan, Chungcheongnam, 31116 Republic of Korea*

[8]*Department of Anatomy and Cell Biology, College of Veterinary Medicine, and Research Institute for Veterinary Science, Seoul National University, Seoul, 08826 Republic of Korea*

[9]*Department of Molecular and Cellular Biochemistry, School of Medicine, Kangwon National University, Chuncheon, Gangwon, 24341 Republic of Korea*

[10]*Department of Biological Sciences, College of Natural Sciences, Kangwon National University, Chuncheon, Kangwon, 24341 Republic of Korea*

[11]*Department of Neurobiology, School of Medicine, Kangwon National University, Chuncheon, Gangwon, 24341 Republic of Korea*

Figure 2A. Supplemental data

The uncut images of blot in Figure 2A is shown

Total Cdk5

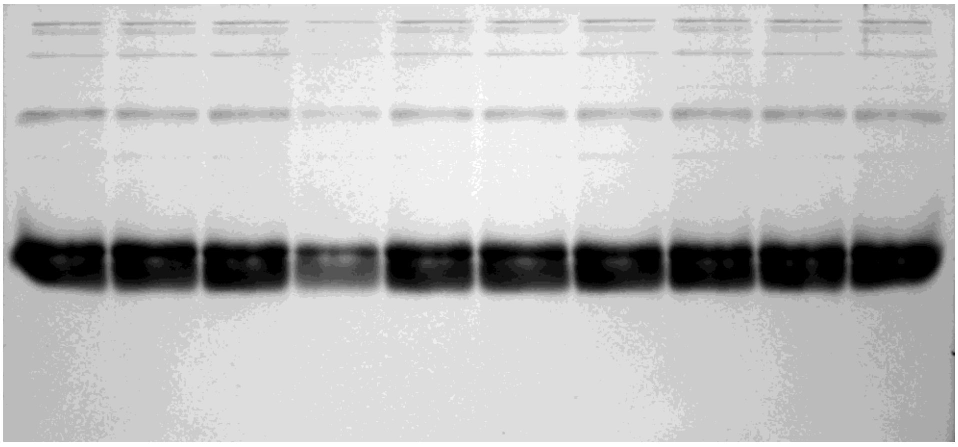

Cytosol Cdk5

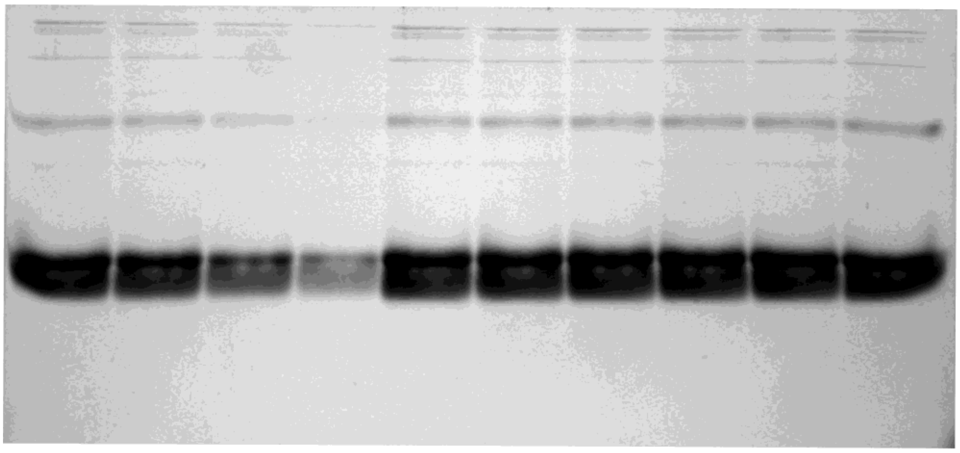

α-tubulin (cytosol fraction)

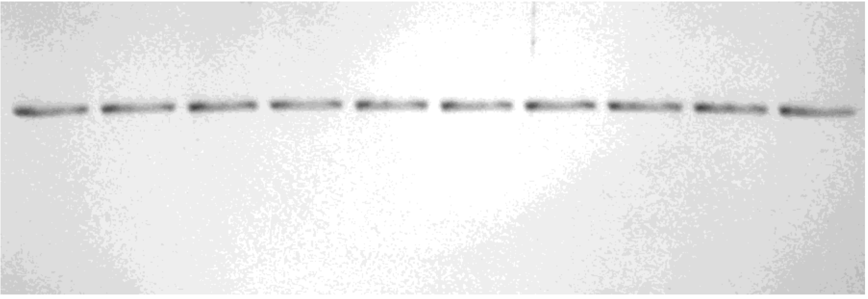

Lamin B (cytosol fraction)

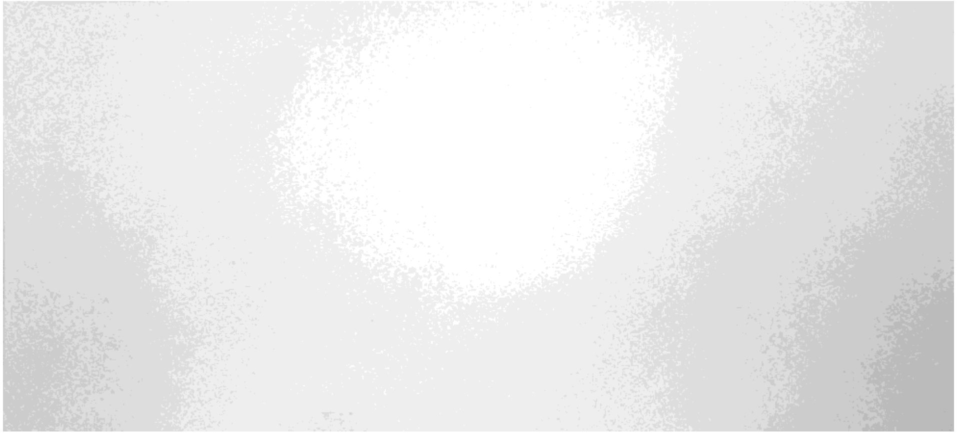

Nucleus Cdk5

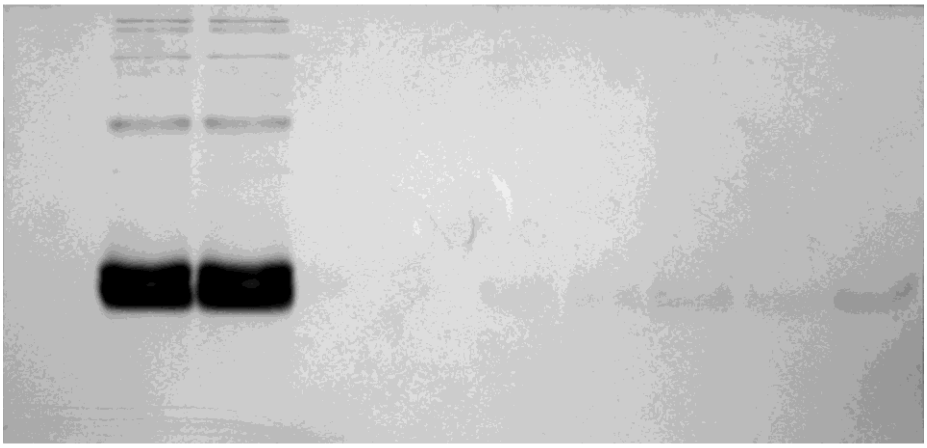

α-tubulin (nucleus fraction)

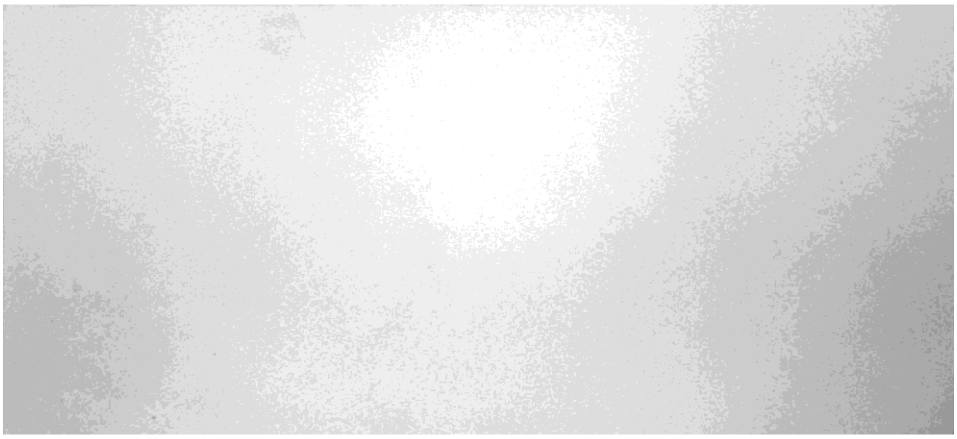

Lamin B (nucleus fraction)

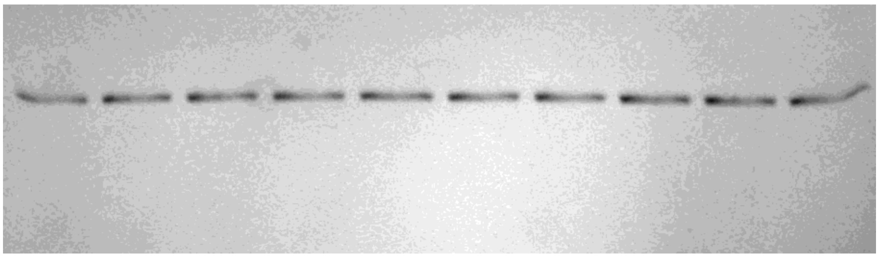

β-actin

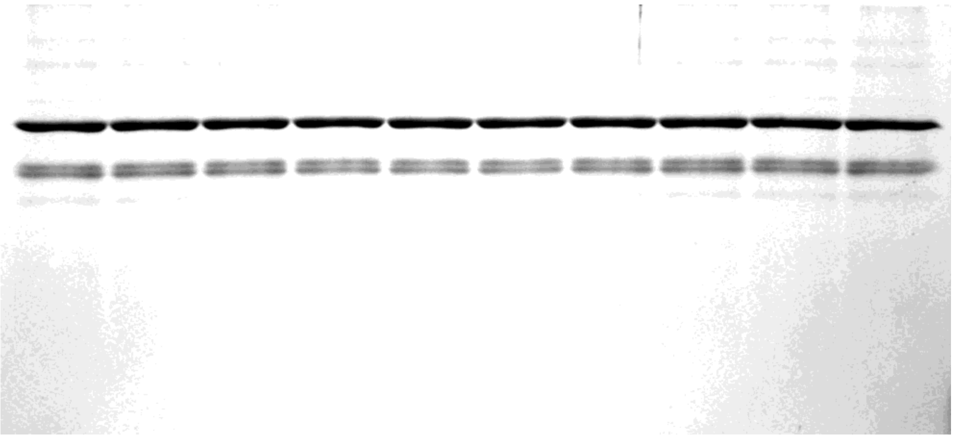

Figure 3. Supplemental data

The uncut images of blot in Figure 3 is shown

p35/p25

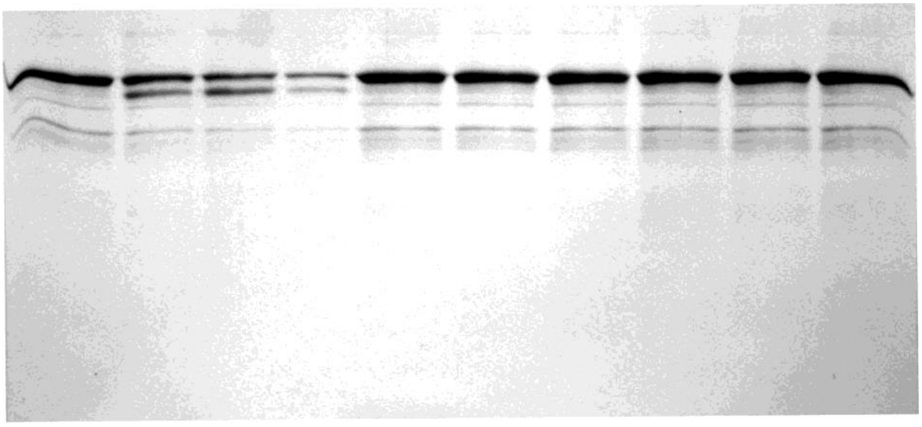

β-actin

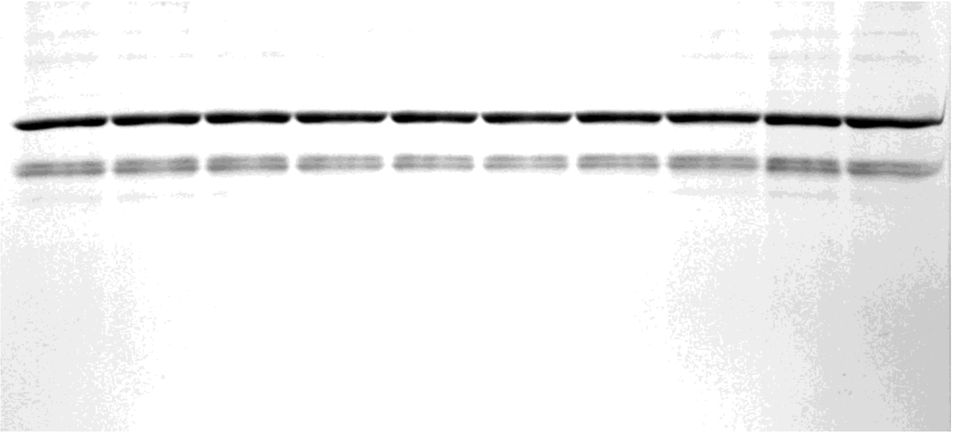

Figure 4A. Supplemental data

The uncut images of blot in Figure 4A is shown

Rb

p-Rb

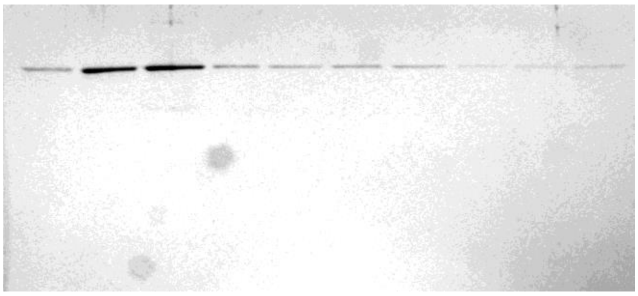

β-actin

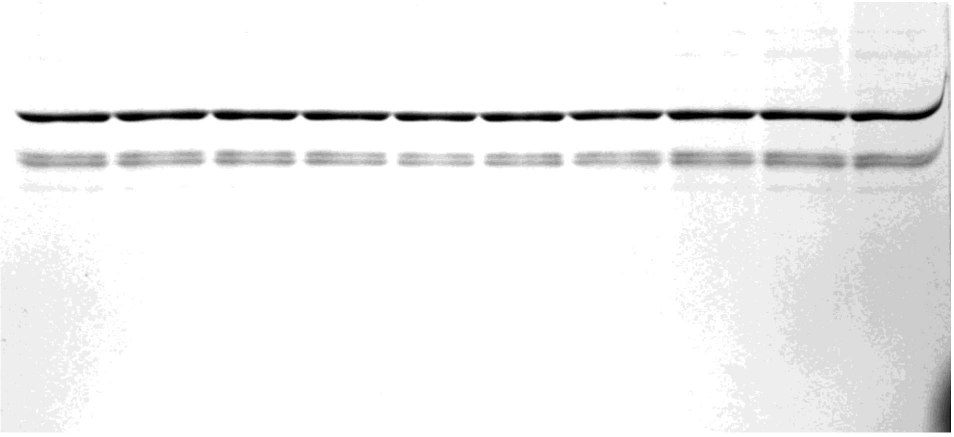

Figure 5A. Supplemental data

The uncut images of blot in Figure 5A is shown

p-53

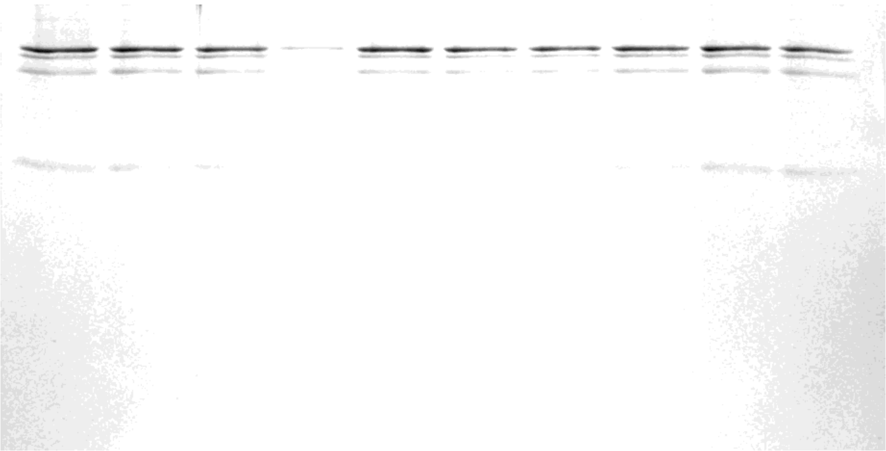

p-p53

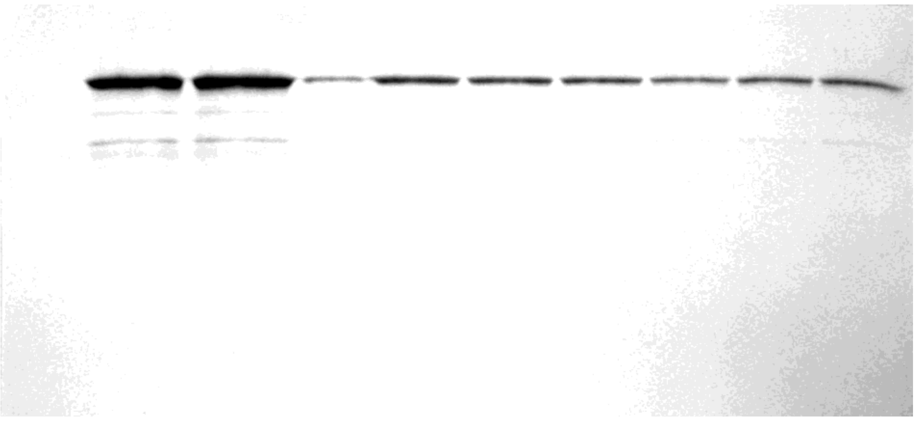

β-actin

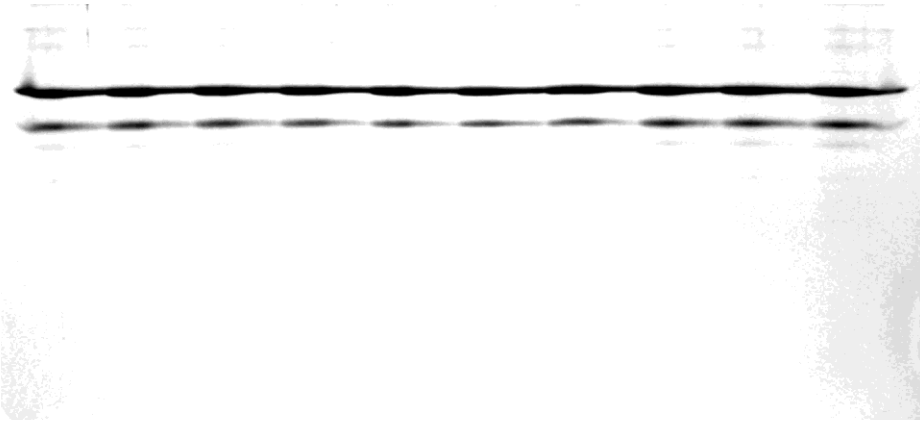

Figure 6. Supplemental data

The uncut images of blot in Figure 6 is shown

Bax

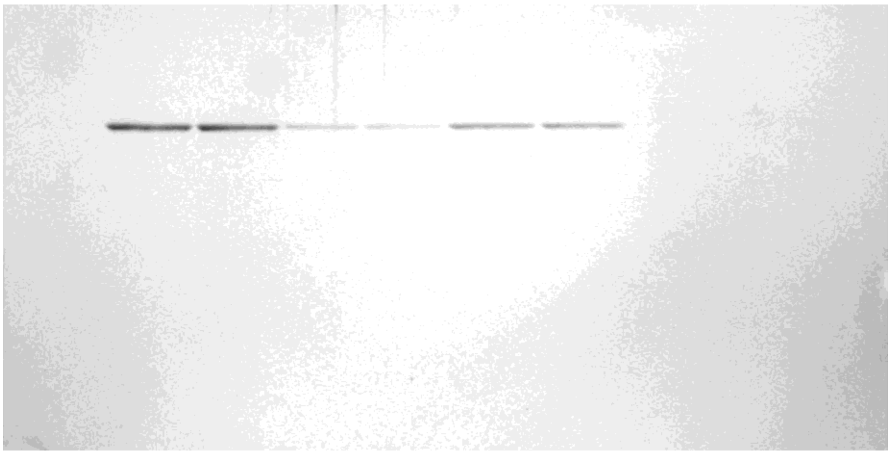

Bcl-2

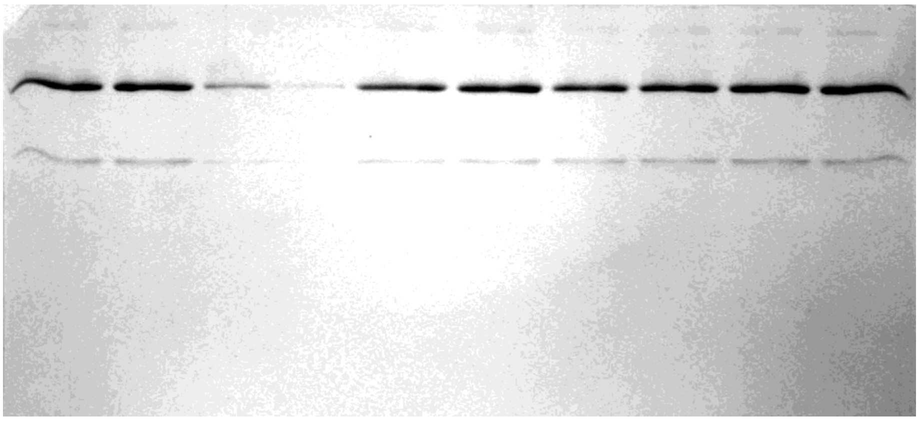

PUMA

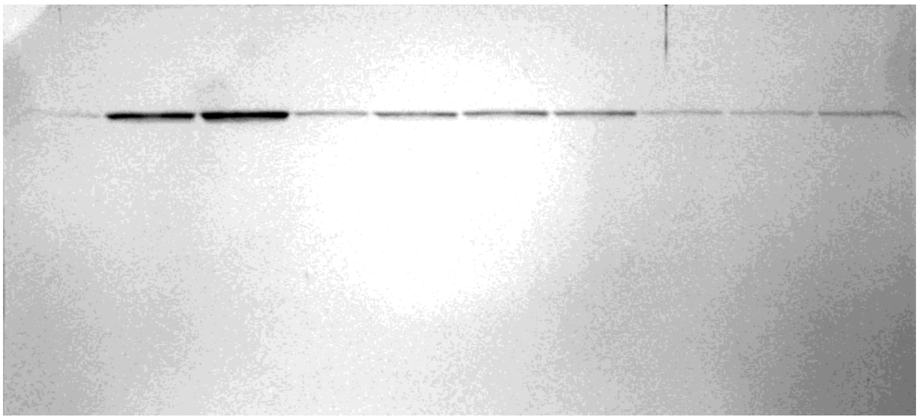

Inactive caspase-3

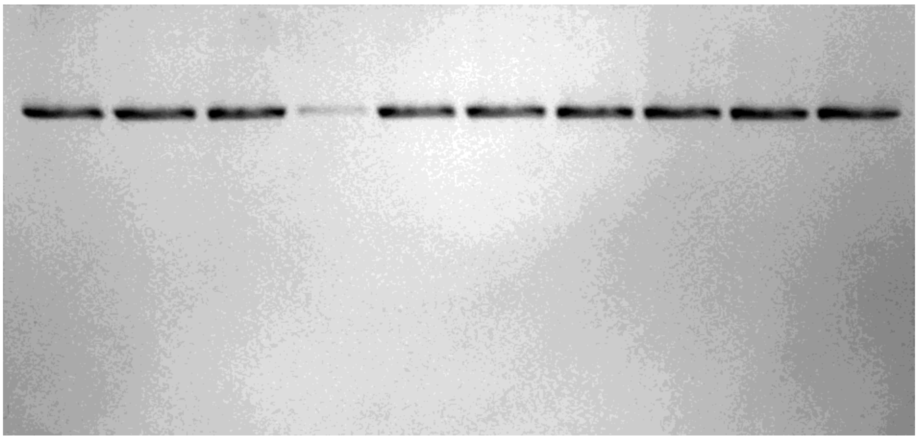

Active caspase-3

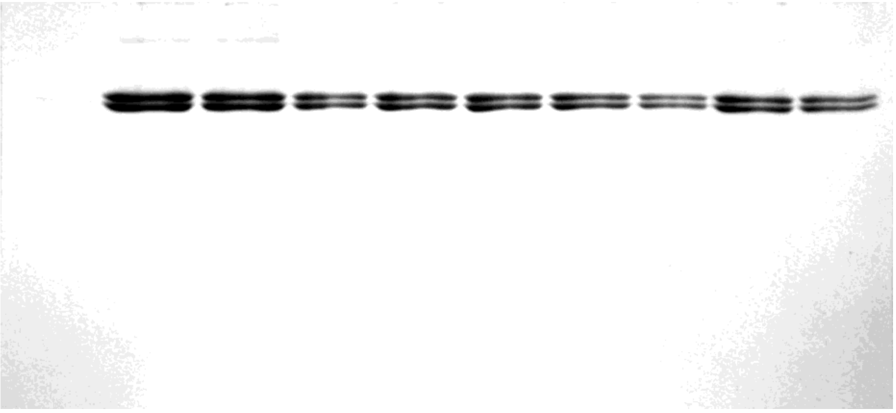

β-actin

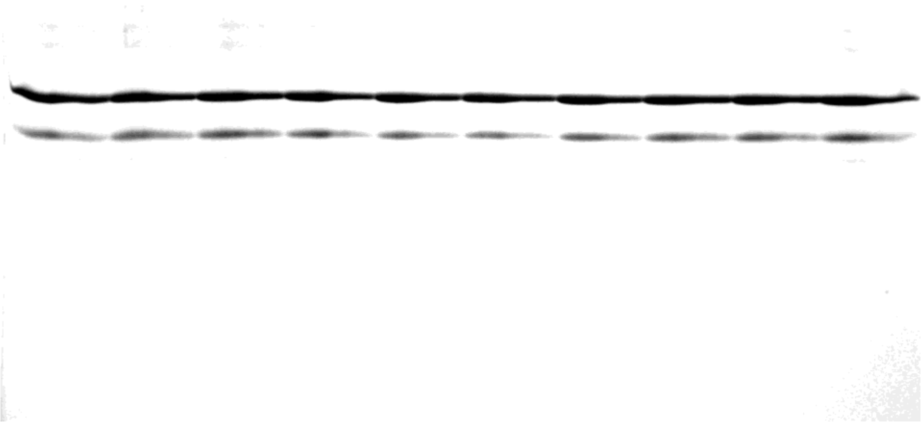

Supplement: Supplementary file 1 — The uncut images of western blot [file 41598_2019_49623_MOESM1_ESM.pdf]
